# In Silico Analysis of the Correlation of KIF2C with Prognosis and Immune Infiltration in Glioma

**DOI:** 10.1155/2022/6320828

**Published:** 2022-03-27

**Authors:** Binfeng Tu, Huali Xiang, Min Li, Fangping Zhong, Meng Fang, Wangjun Yan

**Affiliations:** ^1^Department of Neurosurgery, Jiangxi Cancer Hospital, Jiangxi, China; ^2^Department of Physical Examination Center, Jiangxi Maternal and Child Health Hospital, Jiangxi, China; ^3^Department of Musculoskeletal Surgery of Fudan University Shanghai Cancer Center, Shanghai, China; ^4^Department of Oncology, Shanghai Medical College, Fudan University, Shanghai, China

## Abstract

Glioblastoma (GBM) is one of the most commonly pivotal malignant caners. Numerous reports have revealed the crucial roles of immune infiltration in the initiation and progression of GBM. In this study, we first identified differentially expressed genes (DEGs) in the progression of GBM using CGGA databases. Totally, 156 upregulated DEGs and 251 downregulated DEGs were revealed. By constructing a protein-protein interaction network, KIF2C was identified as a hub gene in GBM. Further analysis revealed an evidently positive association existing in KIF2C expression and the advanced stages of gliomas. Higher expression of KIF2C was in WHO grade IV samples relative to that in grade III and grade II samples. In addition, our results showed that KIF2C was higher in IDH1 wild-type samples than IDH1 mutant glioma samples, in 1p/19q noncodel samples than 1p/19q code glioma samples, and in recurrent samples than primary glioma samples. Moreover, our results showed that higher expression of KIF2C correlated with shorter survival time in both primary and recurrent gliomas and could act as a potential biomarker for the prognosis of GBM. Further analysis demonstrated that higher expression of KIF2C was related to higher levels of endothelial cell, T cell CD8+ naïve, common lymphoid progenitor, T cell CD4+ Th2, T cell CD4+ Th2, macrophage, macrophage M1, T cell CD4+ memory, and T cell CD4+ effector memory, but was related to lower levels of NK cell, B cell plasma, T cell CD4+ Th1, T cell regulatory (Tregs), neutrophil, and T cell NK. We thought this study could provide potential biomarkers for the prediction of prognosis and immune infiltration of gliomas.

## 1. Introduction

Glioblastoma (GBM) derived from neuroepithelium is the widely occurring main malignant neoplasm in the central nervous system (CNS) of adults [1]. More than half of the primary brain neoplasms were GBM [2]. The incidence of GBM increases with age [3]. As per CNS classification standard of the World Health Organization (WHO), GBM belonged to a grade IV neoplasm with astrocytic differentiation and presented high invasiveness, high heterogeneity, and poor prognosis in human CNS [4]. GBM showed no signs of progression in the early stages and was identified as advanced glioma from the onset [4]. Only 3%-5% of patients can survive 5 years after treatment [5]. Lacking indicators for initial diagnosis and corresponding treatments are important reasons of GBM patients' deaths.

Over the past decades, several factors were revealed to affect the tumorigenesis and progression of gliomas [6–9]. For instance, phosphorylating MST4 of ATG4B modulates the activity of autophagy, tumorigenesis, and radioresistance of GBM [6]. Inhibiting telomere protein TRF1 affects the tumorigenesis and development in GBM mouse models and patient-derived xenografts [7]. m^6^A demethylase ALKBH5 preserves the tumorigenesis of GBM stem-like cells via maintaining the expression of FOXM1 and the program of cell proliferation [8]. Activating WNT5A could drive the differentiation and invasive growth of GBM stem cells [9]. However, these previous reports merely focused on a minority of genes in gliomas.

KIF2C, a member of the MT depolymerase family, was reported to take part in mitosis, such as spindle assembly, chromosome condensation, and kinetochore-MT attachment [10]. Several recent studies showed this gene had a crucial role in cancers [10–13]. For example, KIF2C was involved in the regulation of the dynamic and repair of DNA double-strand break in carcinoma cells [14]. KIF2C played as an oncogene in non-small-cell lung carcinoma and exhibited a negative regulation with miR-325-3p [11]. The expression of KIF2C is linked to poor prognostic status of esophageal squamous cell cancer diagnosed in males [15]. However, the roles of KIF2C in GBM remained to be unclear.

In this study, we validated the general DEGs by integrating three CGGA GBM datasets and TCGA cohort and in-depth assessed the functions and pathways of them utilizing Gene Ontology (GO) and Kyoto Encyclopedia of Genes and Genomes (KEGG) analysis. To choose high degree of hub genes, we constructed a PPI (protein-protein interaction) network with the Retrieval of Interacting Genes (STRING) database. Our findings would give an insight to uncovering a probable biomarker for glioma prognosis.

## 2. Materials and Methods

### 2.1. Microarray Data

The present study analyzed three independent databases downloaded from CGGA database (http://www.cgga.org.cn/). Meanwhile, the gene expression data of KIF2C were acquired from TCGA website (https://portal.gdc.cancer.gov/).

### 2.2. Screening the DEGs

To dig out the DEGs with *p* value < 0.05 and ∣log fold change (FC) | >1 in the GSE63678 and GSE17025 datasets, we carried out the limma R package [16]. To further uncover the DEGs in TCGA GBM RNA-sequencing data, we utilized the DESeq2 R package. We acquired the DEGs amid the three datasets with a Venn diagram.

### 2.3. GO and KEGG Enrichment Analyses of the DEGs

The Database for Annotation, Visualization, and Integrated Discovery (DAVID) 16 (version 6.8) offers a whole set of functional annotation tools for researchers to have a better understanding of the biological significance implicated in a large number of genes [17]. clusterProfiler R package was applied to explore GO functional annotation and KEGG of the DEGs.

### 2.4. Construction of PPI Network and Identification of Pivotal Modules

The PPI network was established utilizing STRING (version 10.0) database. Cytoscape (version 3.6.1) is a sort of bioinformatics software applied for the visualization of molecular interplay networks. We employed the plugin molecular complex detection (MCODE) to search the tightly linked areas in the network. Cytoscape was taken to visualize the PPI network, and MCODE was exploited to identify the most evident modules. The standard for selection was degree cutoff = 2, node score cutoff = 0.2, max depth = 100, and *k* score = 2.

### 2.5. Predicting the Significance of Prognosis

We screened genes with the potential prognosis and observed the criteria with a high degree (the number of direct connections between one node and other nodes) among the top 20 hub genes by analysis of TCGA RNA sequencing data of patients. The survminer R package was employed to explore the cutpoint value relevant to the most obvious relationship of patients' status (e.g., survival probability).

To define appropriate items for constructing a nomograph, we conducted univariate and multivariate Cox regression analysis. The forest is applied to display the *p* value, HR, and 95% CI of every variable utilizing the “forestplot” R package. In the light of the data of multivariate Cox proportional hazard analysis, we generated a nomograph to forecast *X*-year overall recurrence ratio. The nomograph supplies a graphical depiction towards these factors, aiming at calculating the recurrence risk of individual patient based on the points related to every risk factor via “rms” R package.

### 2.6. Statistical Analysis

Survival analysis was performed using the Kaplan-Meier method and the log-rank test. R version 4.0.2 and GraphPad 5.0 were used to perform statistical analysis. Differences between groups were evaluated by the Student *t*-test. *p* < 0.05 indicates statistical significance.

## 3. Results

### 3.1. Identification of Progression-Related DEGs in Glioma

**Figure 1:**
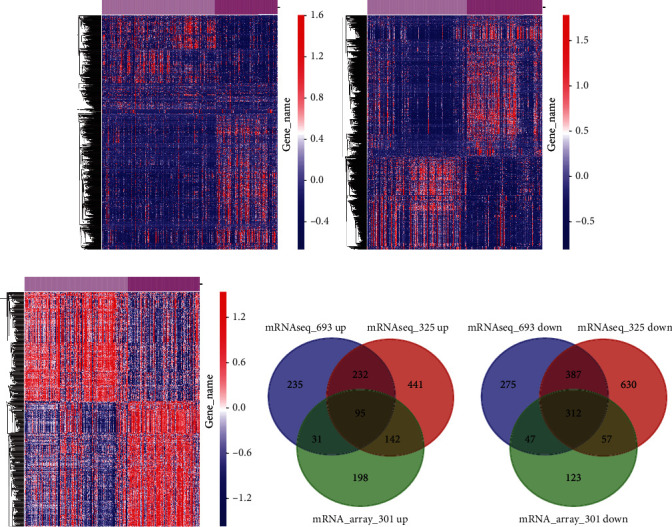
Identification of progression-related DEGs in glioma. The DEGs between low-grade and high-grade glioma samples were identified using (a) mRNA-seq-693 database, (b) mRNA-seq-325 database, and (c) mRNA-array-301 database. (d) Common upregulated and (e) common downregulated genes were identified by Venn diagram.

The present study analyzed three impendent datasets in CGGA databases to discover the DEGs between low-grade and high-grade glioma samples. The genes with *p* < 0.05 and log [FC] > 1 were thought to be DEGs. Finally, 593 DEGs with upregulation and 1021 DEGs with downregulation were found in mRNA-seq-693 database (Figure 1(a)), 910 DEGs with upregulation and 1386 DEGs with downregulation were found in mRNA-seq-325 database (Figure 1(b)), and 496 DEGs with upregulation and 539 DEGs with downregulation were found in mRNA-array-301 database (Figure 1(c)). Finally, a total of 95 common upregulated (Figure 1(d)) and 312 common downregulated genes (Figure 1(e)) were identified in high-grade compared to low-grade glioma samples. These genes were presented by a heat map.

Figure 1 presents the general DEGs screened by Venn diagram amid three abovementioned datasets. 407 DEGs including 156 genes with increasing expression and 251 genes with decreasing expression were unearthed.

### 3.2. Bioinformatics Analysis of DEGs

**Figure 2:**
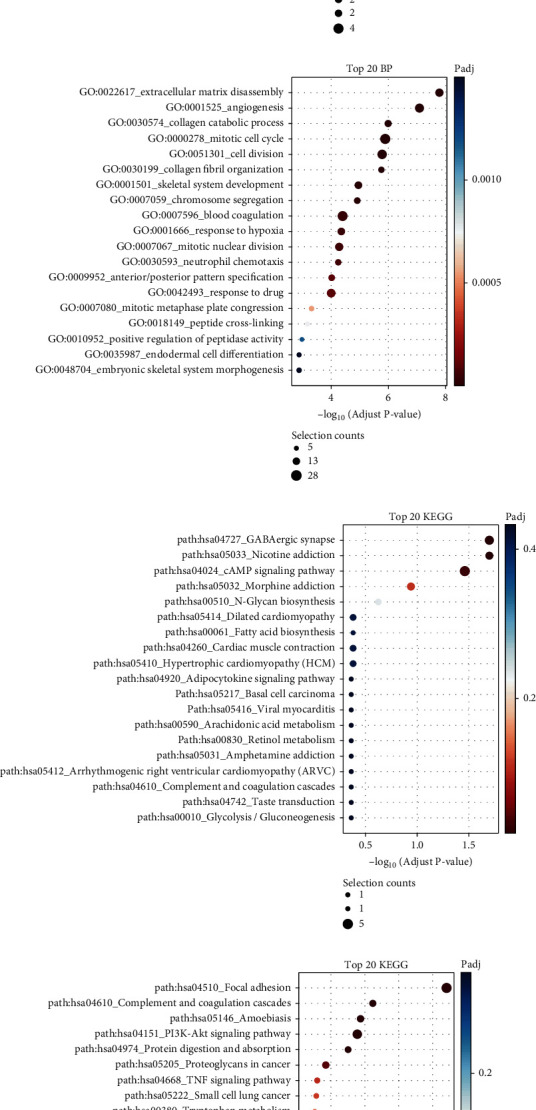
Bioinformatics analysis of DEGs. (a, b) The GO analysis of upregulated and downregulated genes in gliomas. (c, d) The KEGG analysis of upregulated and downregulated genes in gliomas.

To explore possible function of these DEGs, we performed GO and KEGG pathway analysis.  Figure 2 depicts that genes with upregulation were primarily enriched in synaptic transmission, potassium ion transmembrane transport modulation, synapse assembly positive modulation, somatostatin signaling pathway, response to nutrient, and progesterone metabolic process (Figure 2(a)). Genes with downregulation were chiefly enriched in extracellular matrix organization and disassembly, angiogenesis, collagen catabolic process, mitotic cell cycle, cell division, collagen fibril organization, skeletal system development, chromosome segregation, blood coagulation, response to hypoxia, mitotic nuclear division, and neutrophil chemotaxis (Figure 2(b)).

**Figure 3:**
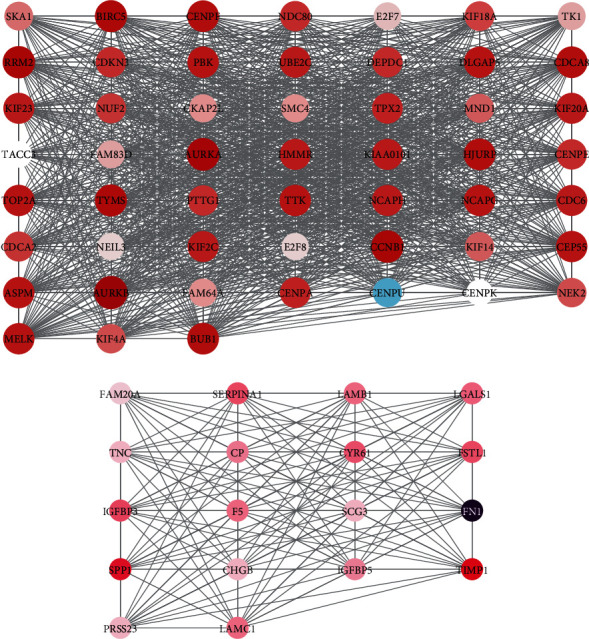
Construction of PPI network in gliomas. (a) Hub network 1 with 52 genes was constructed. (b) Hub network 2 with 18 genes was constructed.

Figure 3 demonstrates that the DEGs with increasing expression pivotally participated in neuroactive ligand-receptor interplay, GABAergic synapse, nicotine and morphine addiction, cAMP signaling pathway, N-glycan biosynthesis, cardiac muscle contraction, hypertrophic cardiomyopathy (HCM) and dilated cardiomyopathy, and fatty acid biosynthesis (Figure 2(c)). The DEGs with reduced expression were primarily enriched in PI3K-Akt, TNF and p53 signaling pathways, protein digestion and absorption, proteoglycans in carcinoma, small cell lung carcinoma, tryptophan metabolism, and bladder carcinoma (Figure 2(d)).

### 3.3. To Construct PPI Network and Identify the Hub Genes

We employed STRING database to assess the interplay amid the DEGs. The extraction and visualization of these DEGs were completed by Cytoscape software. The PPI network composed of 284 nodes and 3697 edges is presented in Figure 3(a) after removal of isolated nodes (Supplementary Figure 1).

The MCODE plugin identifies an important connection module. Two hub PPI networks were identified. Hub network 1 included 52 genes (DLGAP5, PBK, KIF2C, CEP55, TACC3, CENPA, HJURP, CENPF, CENPE, KIF18A, KIF14, MND1, SMC4, KIF20A, NCAPH, NCAPG, BIRC5, ASPM, CDCA8, NDC80, AURKB, CDKN3, AURKA, TPX2, CENPU, CENPK, NUF2, BUB1, FAM83D, NEK2, KIF4A, E2F7, FAM64A, E2F8, CDCA2, TOP2A, TK1, TYMS, HMMR, NEIL3, PTTG1, KIAA0101, SKA1, TTK, CDC6, RRM2, UBE2C, DEPDC1, CCNB1, CKAP2L, MELK, and KIF23) (Figure 3(a)), and hub network 2 included 18 genes (SERPINA1, FAM20A, FN1, TIMP1, F5, IGFBP5, SPP1, TNC, SCG3, CHGB, LGALS1, PRSS23, CYR61, LAMB1, IGFBP3, LAMC1, FSTL1, and CP) (Figure 3(b)).

The dysregulation of hub DEGs was correlated to the survival time in patients with glioma.

**Figure 4:**
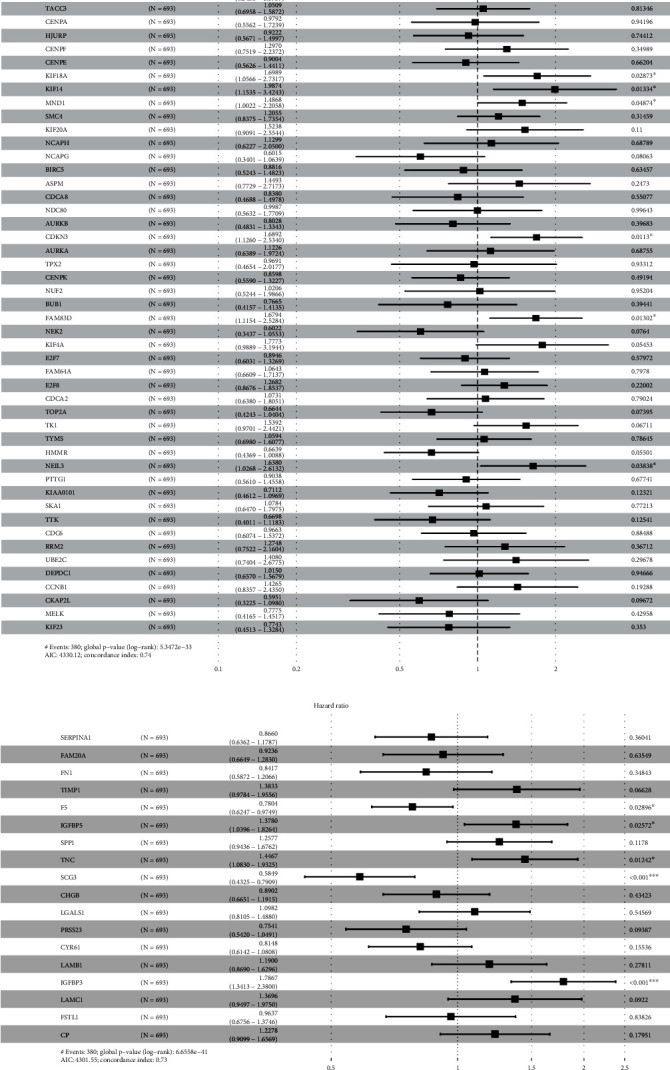
The forest map analysis of the correlation between hub DEG expression and survival time in patients with glioma. (a) Forest map showed 8 hub genes in hub network 1 were related to the poor prognosis of gliomas, including PBK, KIF2C, CENPE, KIF14, MND1, FAM83D, NEIL3, and CDKN3. (b) Forest map showed 5 hub genes in hub network 2 were related to the poor prognosis of gliomas, including F5, IGFBP5, TNC, SCG3, and IGFBP3.

Then, a forest map was produced to analyze the correlation between hub DEGs and OS analysis. After calculating the hazard ratio and confidence interval, we observed that 8 hub genes in hub network 1 were related to the poor prognosis of gliomas, including PBK, KIF2C, CENPE, KIF14, MND1, FAM83D, NEIL3, and CDKN3 (Figure 4(a)). In addition, 5 hub genes in hub network 2 were related to the poor prognosis of gliomas, including F5, IGFBP5, TNC, SCG3, and IGFBP3 (Figure 4(b)).

**Figure 5:**
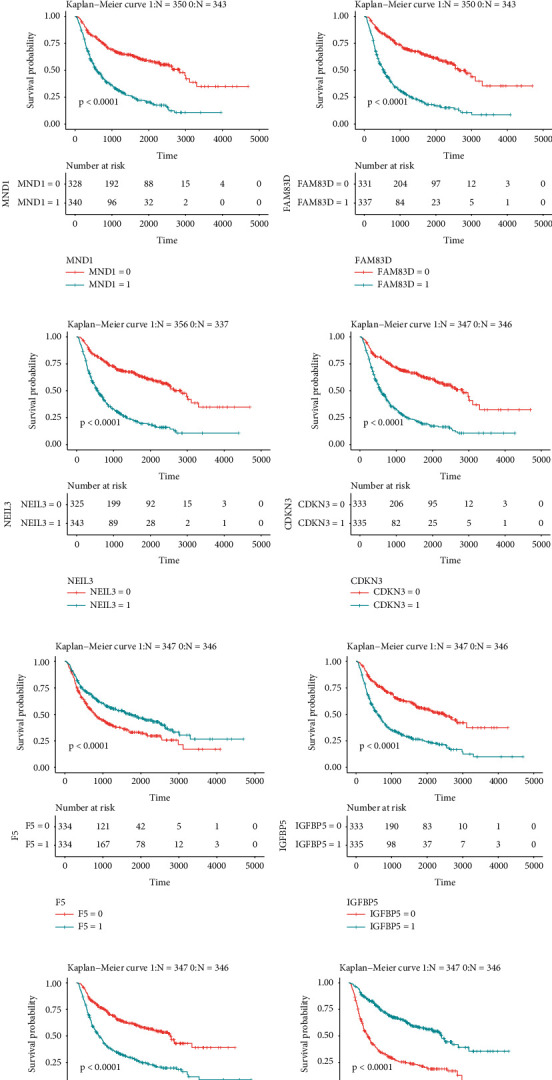
The dysregulation of hub DEGs in hub network was correlated with the survival time in patients with glioma. Higher expression level of (a) PBK, (b) KIF2C, (c) CENPE, (d) KIF14, (e) MND1, (f) FAM83D, (g) NEIL3, (h) CDKN3, (i) F5, (j) IGFBP5, (k) TNC, (l) SCG3, and (m) IGFBP3 exhibited a significant association with shorter OS time in glioma patients.

On the basis of CGGA data, we have drawn Kaplan-Meier plots and the data demonstrated the impacts of these top hub genes on survival probability.  Figure 5 reveals that the higher expression level of PBK (Figure 5(a)), KIF2C (Figure 5(b)), CENPE (Figure 5(c)), KIF14 (Figure 5(d)), MND1 (Figure 5(e)), FAM83D (Figure 5(f)), NEIL3 (Figure 5(g)), and CDKN3 (Figure 5(h)) in hub network 1 exhibited a significant association with shorter OS time in glioma patients. Higher expression level of IGFBP5 (Figure 5(i)), TNC (Figure 5(j)), and IGFBP3 (Figure 5(k)) was shown in hub network 2, and the lower expression level of F5 (Figure 5(l)) and SCG3 (Figure 5(m)) was dramatically related to shorter OS time in glioma patients.

### 3.4. Evaluation of the Correlation of KIF2C Expression and Clinicopathologic Characteristics

**Figure 6:**
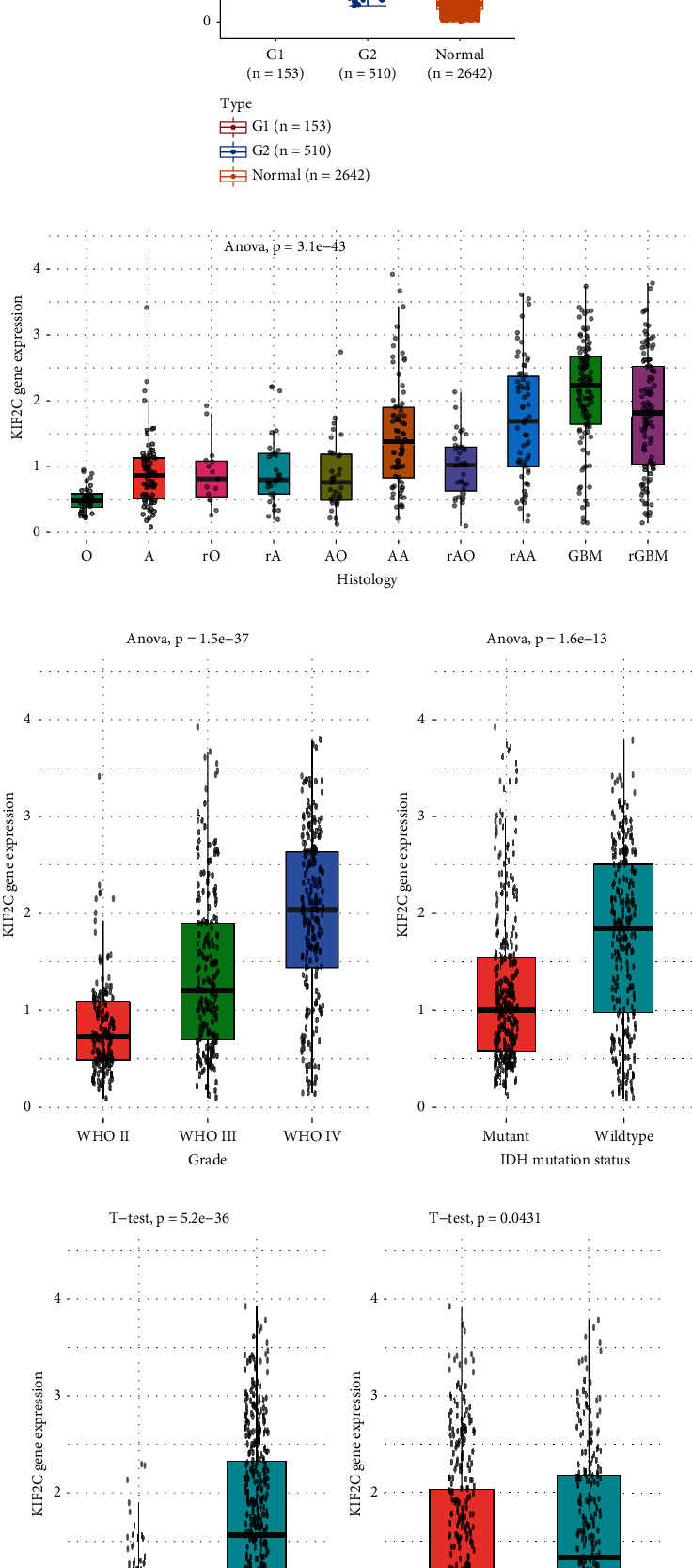
Evaluation of the correlation of KIF2C expression and clinicopathologic characteristics. (a) KIF2C was differently expressed in multiple histology of gliomas. (b) KIF2C expression had an obviously positive correlation with the advanced grade gliomas. (c) Higher expression of KIF2C was in WHO grade IV samples relative to that in grade III and grade II samples and in WHO grade III samples compared to that in grade II samples. (d) KIF2C was higher in IDH1 wild-type samples than IDH1 mutant glioma samples. (e) KIF2C was higher in 1p/19q noncodel samples than 1p/19q code glioma samples. (f) KIF2C was higher in recurrent samples than primary glioma samples (Figure 8(f)).

Among these hub genes, KIF2C showed the most significant correlation to prognosis, which had been reported to be a key role in cell cycle regulation. We chose KIF2C here to validate the links existing in the expression and its clinicopathological values in CGGA cohort. The data showed different degrees of KIF2C expression in multiple histology of gliomas (Figure 6(a)). We found an evidently positive association existing in KIF2C expression and the advanced stages of gliomas. Further analysis also confirmed that KIF2C expression had an obvious positive correlation with the advanced grades of gliomas (Figure 6(b)). Higher expression of KIF2C was in WHO grade IV samples relative to that in grade III and grade II samples (Figure 6(c)), and higher expression of KIF2C was in WHO grade III samples compared to that in grade II samples (Figure 6(c)). In addition, our results showed that KIF2C was higher in IDH1 wild-type samples than IDH1 mutant glioma samples (Figure 6(d)), and KIF2C was higher in 1p/19q noncodel samples than 1p/19q code glioma samples (Figure 6(e)). Finally, KIF2C was higher in recurrent samples than primary glioma samples (Figure 6(f)).

### 3.5. Higher Expression of KIF2C Was Correlated with Shorter Survival Time in Glioma

**Figure 7:**
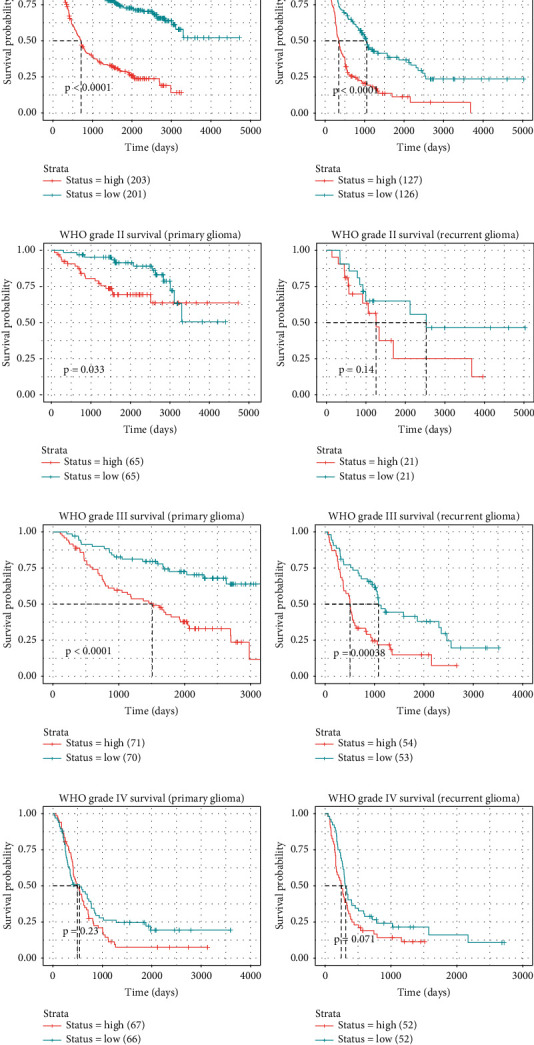
Higher expression of KIF2C was correlated with shorter survival time in glioma. (a, b) Higher expression of KIF2C correlated to shorter survival time in both primary and recurrent gliomas. (c, d) The correlation between KIF2C expression and survival time in primary and recurrent grade II gliomas. (e, f) The correlation between KIF2C expression and survival time in primary and recurrent grade III gliomas. (g, h) The correlation between KIF2C expression and survival time in primary and recurrent grade IV gliomas.

Survival curve of the gene was also generated to evaluate the correlation between KIF2C expression and survival time in primary and recurrent gliomas using CGGA data (Figure 7). Our results showed that higher expression of KIF2C correlated with shorter survival time in both primary and recurrent gliomas (Figures 7(a) and 7(b)). In grade II glioma patients, we observed that higher expression of KIF2C was correlated with shorter survival time in primary (Figure 7(c)), but not with recurrent gliomas (Figure 7(d)). In grade III glioma patients, we observed that higher expression of KIF2C presented a correlation with shorter survival time in both primary and recurrent gliomas (Figures 7(e) and 7(f)). However, no significant correlation between KIF2C expression and survival time was in grade IV gliomas (Figures 7(g) and 7(h)).

### 3.6. Construction of Nomogram for KIF2C in Gliomas

**Figure 8:**
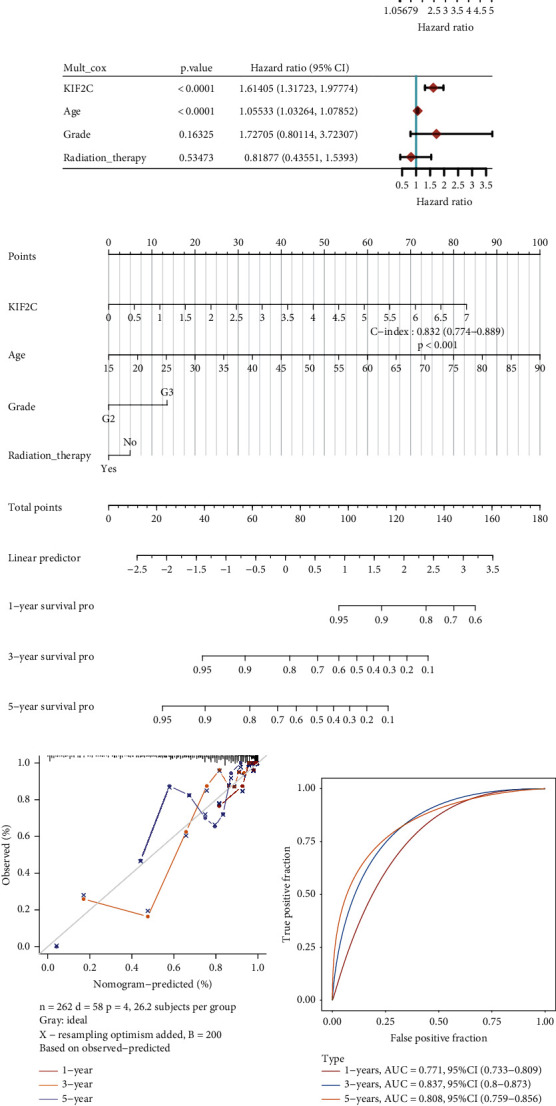
Construction of nomogram for KIF2C in gliomas. (a, b) Univariate and multivariate Cox regression analysis showed KIF2C expression was related to prognosis in gliomas. (c) Nomogram to predict the correlation between KIF2C expression and overall survival in gliomas. (d) Calibration curve for the overall survival nomogram model. (e) The AUC for OS prediction was calculated.

In order to validate the assumption that KIF2C was an independent factor for prognosis of gliomas patients, we conducted univariate and multivariate Cox regression analysis. The result showed that KIF2C expression, age, grade, and radiation therapy were evident factors that were related to glioma prognosis (Figure 8(a)). In addition, multi-Cox regression analysis data indicated that only KIF2C expression and age displayed an association with glioma patient prognosis (Figure 8(b)).

We in-depth established a monograph containing the prognostic factors, such as KIF2C expression, age, and radiation therapy, to supply a quantitative base to forecast the possibility of one-year, three-year, and five-year OS in glioma patients for clinicians (Figure 8(c)). What is more, our data indicated that a nomogram could better evaluate one-year, three-year, and five-year OS compared to Kaplan-Meier (Figure 8(d)). Next, the AUC for OS prediction was calculated. The AUC of the ROC curve reached 0.771 at 1 year, 0.837 at 3 years, and 0.808 at 5 years (Figure 8(e)).

### 3.7. KIF2C Dysregulation Was Associated with Immune Infiltration in Gliomas

**Figure 9:**
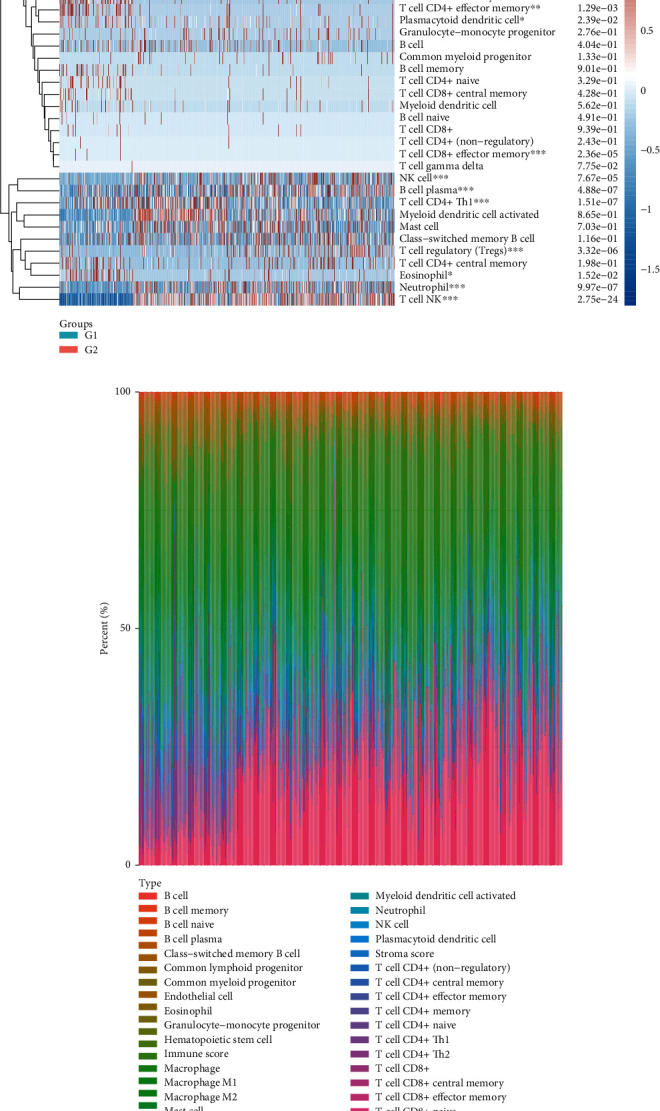
KIF2C dysregulation was associated with immune infiltration in gliomas. (a, b) The correlation between KIF2C expression and immune infiltration in gliomas was analyzed using the Xcell dataset.

In the present study, we analyzed the correlation between KIF2C expression and immune infiltration in gliomas using the Xcell dataset. As presented in Figure 9, we revealed that KIF2C high expression was related to higher levels of endothelial cell, T cell CD8+ naïve, common lymphoid progenitor, T cell CD4+ Th2, T cell CD4+ Th2, macrophage, macrophage M1, T cell CD4+ memory, and T cell CD4+ effector memory (Figures 9(a) and 9(b)). However, we found that KIF2C high expression was related to lower levels of NK cell, B cell plasma, T cell CD4+ Th1, T cell regulatory (Tregs), neutrophil, and T cell NK (Figures 9(a) and 9(b)).

## 4. Discussion

GBM is a widespread and precarious malignant brain neoplasm, of which symptoms include abdominal distension, pelvic pain, difficulty in eating, and frequent urination [18]. As a type of highly invasive tumors, GBM accounts for the most among main malignant neoplasms in the brain, ranging from 77% to 80%. Amid GBM patients, the one-year survival rate is approximately 50%, while the three-year survival rate is only 10% [3]. Hence, there is an urgent need to identify new favorable clinical test indicators for GBM diagnosis and prognosis. Early diagnosis (I/II) of GBM is very difficult since most symptoms of GBM are nonspecific. In the past few years, with the advancement of technologies including sequencing and high-throughput DNA microarray analysis, large volumes of data are available online. It is urgent and meaningful for us to screen new effective biomarkers for glioblastoma through a wider range of data.

The present study analyzed three independent datasets in CGGA databases to define the DEGs between low-grade and high-grade glioma samples. Finally, 407 DEGs associated with glioma development were uncovered. Among them, 156 genes were increased and 251 genes were reduced. Bioinformatics analysis showed that genes with upregulation primarily participated in synaptic transmission and cAMP signaling pathway. Of note, our analysis revealed that upregulated genes were related to multiple key signaling in carcinoma progression, such as angiogenesis, mitotic cell cycle, and PI3K-Akt, TNF, and p53 signaling pathways.

The PI3K (PI3 kinase) family displays an intricate role in biological process, such as metabolism [19, 20]. In most HGG brain neoplasms (including GBM), the PI3K pathway is motivated and its activation participated in the neoplasm transition from low grade to high grade [21]. p53 functions importantly in cell cycle regulation, DNA repair, apoptosis, senescence, angiogenesis, and metabolism, forming a greatly complicated signaling network [22]. The changes in the tumor inhibitor gene p53 are the most common in primary GBM and secondary GBM, attaining 25-30% and 60-70%, separately [23].

In order to identify the hub genes involved in glioma progression, we constructed PPI network analysis, and two hub PPI networks were identified. Hub network 1 included 52 genes, and hub network 2 included 18 genes. Also, we observed 13 hub genes were related to the poor prognosis of gliomas, including PBK, KIF2C, CENPE, KIF14, MND1, FAM83D, NEIL3, CDKN3, F5, IGFBP5, TNC, SCG3, and IGFBP3. Of note, the functional importance of several DEGs had been demonstrated in gliomas in previous reports. For example, PBK, a serine/threonine kinase, belonged to the family of mitogen-activated protein kinase kinase (MAPKK), which participated in modulating cell proliferation, metastasis, and autophagy [24]. In gliomas, Dong et al. found that PDZ-binding kinase (PBK) was highly expressed in GBM and also exhibited a negative association with survival status in comparison with brain normal samples [25]. Therefore, it is a potential factor for GBM prognosis and a prospective target for GBM treatment. KIF14, as the mitotic kinesin superfamily protein, is required for cytokinesis and chromosome segregation [26]. Increased expression of KIF14 is associated with a great quantity of human carcinomas. Inhibiting KIF14 would hinder the cell growth of neoplasms and induce apoptosis in human GBM [27]. Endonuclease VIII-like 3 (Neil3) is one part of the five DNA glycosylases in mammals and was used for recognition and removal of oxidized bases and initiation of the base excision repair (BER) pathway [28]. Previous studies have shown that NEIL3 overexpression exhibited an association with genomic changes and low survival in some types of human carcinomas, such as glioma [29]. rs12645561 in NEIL3 were associated with developing GBM in the Han Chinese population [30]. IGFBP5 rather than IGFBP3 overexpression displayed a correlation with the histologic grade of human diffuse glioma [31]. IGFBP5 mediated an increase of cell invasion but an inhibition of cell proliferation via the EMT and Akt signaling pathway in pleomorphic cells of GBM [32]. IGFBP-3 is thought to be a powerful predictor of survival in newly diagnosed GBM patients [33]. Impediment of neoplasm growth after depleting IGFBP3 is regarded as a promising strategy for glioma treatment [34]. Overexpressing IGFBP-3 led to the promotion of cell proliferation, colony formation, and G1/S phase transformation in U87MG and U251MG cells [35].

KIF2C was chosen for subsequent studies amid the selected hub genes. A previous study showed KIF2C is a marker for prognosis in human gliomas [36]. In this study, we found KIF2C expression level presented a great positive correlation with the advanced stages of gliomas. Our results showed that higher expression levels of KIF2C were correlated with shorter overall and disease-free survival time in OS. Furthermore, TCGA database analysis revealed that the model applied for predicting OS and DFS was efficient. The model-based nomogram shows a deep impression of performance and clinical use.

Emerging studies had demonstrated tumor-infiltrating lymphocytes (TILs) could affect the immune response and tumor initiation and progression. TILs had also been found to be enriched in GBM. Very interestingly, several genes had been reported to modulate the immune infiltrating. For example, LRRK2 correlates with macrophage infiltration in GBM [37]. Meanwhile, CD163 was also associated with immune infiltration in glioblastoma multiforme [38]. Moreover, a recent study showed TUBA1C expression was positively related to infiltration levels of multiple immune cells, such as CD8 T+ cells and neutrophils [39]. However, the correlation between KIF2C and immune infiltration remained to be unclear. Our analysis for the first time demonstrated that higher expression of KIF2C was related to higher levels of endothelial cell, T cell CD8+ naïve, common lymphoid progenitor, T cell CD4+ Th2, T cell CD4+ Th2, macrophage, macrophage M1, T cell CD4+ memory, and T cell CD4+ effector memory, but was related to lower levels of NK cell, B cell plasma, T cell CD4+ Th1, T cell regulatory (Tregs), neutrophil, and T cell NK, indicating that KIF2C may serve as a predictive marker for immune therapy response in GBM.

Several limitations should be noted. First, the functional roles of KIF2C were not confirmed using experimental methods. Gain- or loss-of-function assays should be performed in the future study. Second, despite the fact that two independent datasets were used to explore the clinical significance of KIF2C in GBM, further validation using clinical samples is still needed. Finally, the association between KIF2C expression and immune infiltration was determined using bioinformatics analysis, thus lacking more confirmation.

Collectively, our current study identified 407 progression-related DEGs in gliomas. Bioinformatics analysis showed that these DEGs were related to cAMP signaling, mitotic cell cycle, and PI3K-Akt and p53 signaling pathways. Among these DEGs, 13 hub genes were demonstrated to be associated with the poor prognosis of gliomas, including PBK, KIF2C, CENPE, KIF14, MND1, FAM83D, NEIL3, CDKN3, F5, IGFBP5, TNC, SCG3, and IGFBP3. It is found that the DEGs, especially KIF2C, may provide diagnostic and prognostic value for GBM. In the future study, further research is required to unclose the underlying mechanisms and develop novel therapeutic strategies for GBM.

## Data Availability

The present study analyzed three independent databases downloaded from CGGA database. Meanwhile, the gene expression data of KIF2C were acquired from TCGA website.

## Supplementary Materials

Supplementary Materials Supplementary Figure 1: PPI network differential genes of DEGs.
