# Supplementary figures and images for: In Silico Analysis of the Correlation of KIF2C with Prognosis and Immune Infiltration in Glioma
Source: Comput Math Methods Med. 2022 Mar 27;2022:6320828. doi: 10.1155/2022/6320828 (PMC8977321; doi:10.1155/2022/6320828)

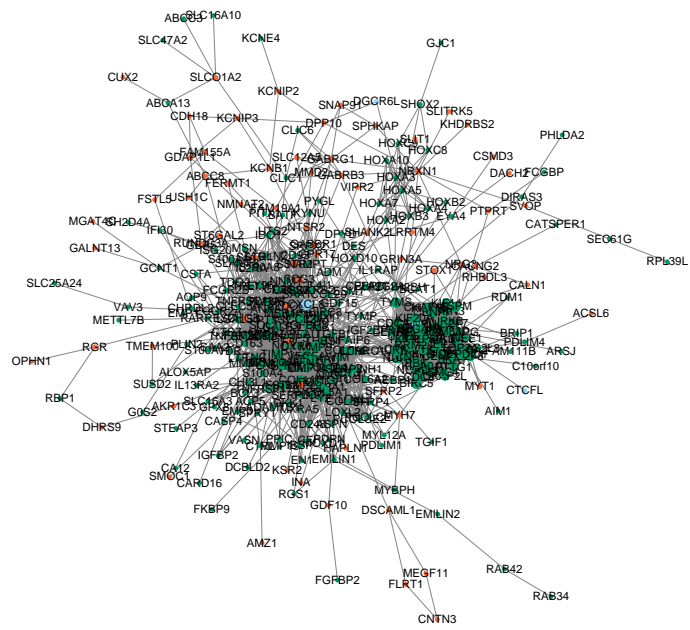

Supplement: Supplementary Materials — Supplementary Figure 1: PPI network differential genes of DEGs. [file 6320828.f1.pdf]
